# Average Hourly Concentrations of Air Contaminants in Selected Urban, Town, and Rural Sites

**DOI:** 10.1007/s00244-019-00627-8

**Published:** 2019-04-10

**Authors:** Robert Cichowicz, Artur Stelęgowski

**Affiliations:** Faculty of Civil Engineering, Architecture and Environmental Engineering, Lodz University of Technology, Lodz, Poland

## Abstract

**Abstract:**

The inhabitants of cities, towns, and villages are exposed to different levels of air pollution, which also vary throughout the day. Information regarding episodes of poor and good air quality enables planning to mitigate the risks and maximize the benefits of spending time outdoors. In this work, an analysis was made of the state of air quality 2012–2016, using data gathered from automatic measuring stations located in five cities (> 50,000 inhabitants), five towns (5000–50,000 inhabitants), and five villages (< 5000 inhabitants) in five neighboring provinces in central Poland, in Central Europe. The monitoring stations were designated as “city background”, “town background”, and “rural background”. More than 3 million pieces of data were collected from 15 monitoring stations. This allowed the average daily changes in the concentration of air pollutants (NO_2_ and NO_x_, O_3_, SO_2_, CO, PM_10_, PM_2.5_, C_6_H_6_) to be determined, depending on the type of station and the size of the settlement unit in both winter periods and summer periods. As a result, the most and least favorable hours in terms of levels of air pollution were identified. This information could help to inform air quality management in modern cities, towns, and villages and to improve the quality of life, particularly among those most susceptible to the negative effects of air pollution, such as the elderly and children.

**Graphical Abstract:**

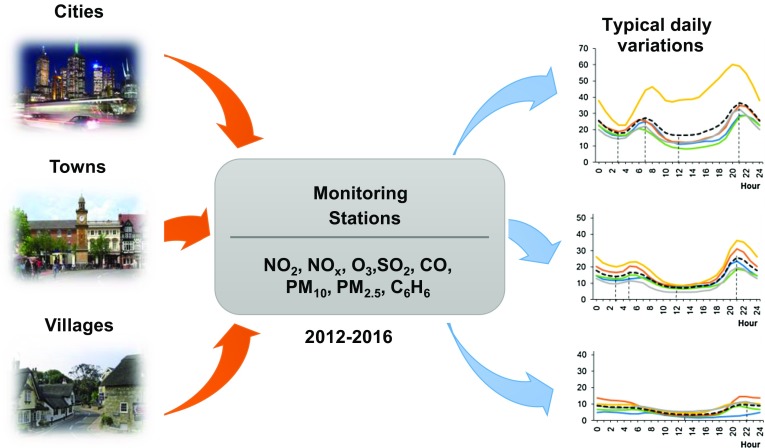

## State-of-the-Art

Worldwide air quality poses a risk both to human health and the wider environment (Agudelo-Castañeda et al. 2019; Ramírez et al. 2019; Oliveira et al. 2019). Studies on air pollution focus not only on inhabited areas (Cichowicz et al. 2017; Cichowicz 2018; Pérez et al. 2010) but also on agricultural and undeveloped areas, which are used as background measurements (Hagenbjörk et al. 2017; Putaud et al. 2004; Zheng et al. 2010). In many countries, including in Europe, air quality assessments use measurements performed by automatic measuring stations (Agudelo-Castaneda and Teixeira 2014; Malley et al. 2018; Wielgosiński et al. 2018) and air quality modeling (Cichowicz and Stelegowski 2018a; Inness et al. 2013; Menut et al. 2012). Usually, the average annual, seasonal, and monthly concentrations of air pollutants are analyzed (Cichowicz et al. 2017; Guerreiro et al. 2010; Malley et al. 2018). Much less information is available regarding daily fluctuations (Liu et al. 2014; Zhao et al. 2009). Daily variations in air pollution occur due to the high variability of sociophysical and chemical parameters during the day (Menut et al. 2012; Pérez et al. 2010). These changes depend largely on the day of the week (work days or weekends) (Feng et al. 2014; Chaloulakou and Mavroidis 2002) and the season (Vouitsis et al. 2015). The average hourly concentrations of air pollutants are usually analyzed only for cities (Agudelo-Castaneda and Teixeira 2014; Vouitsis et al. 2015) and in the vicinity of busy roads (Moreno et al. 2013; Pérez et al. 2010). There are few air-quality analyses on other types of area, especially for settlement units of different sizes, and those focus on average annual and seasonal pollution levels (Hagenbjörk et al. 2017; Malley et al. 2018; Putaud et al. 2004; Tecer and Tagil 2014) and only occasionally daily variations (Zheng et al. 2010).

## Outdoor and Indoor Air Pollution

Concentrations of air pollutants depend on city density and on urban planning, and the level of industrialization also has an impact (Landim et al. 2018; León-Mejía et al. 2018; Saikia et al. 2018). The daily activity patterns of people, including time spent at home, working hours, and travel, are specific to particular areas and fluctuate during the day. The emission of air pollutants associated with such activities, and the concentrations of pollutants in the ambient air therefore vary (Cichowicz et al. 2017; Fenger 1999; Nicolai et al. 2003). Although people spend only 10–20% of their days outdoors, it is still important to provide information on air quality, especially for those most susceptible to the negative effects of air pollution, such as children, the elderly, and people with asthma and respiratory illnesses (Künzli et al. 2000; WHO 2000, 2005). Such information makes it possible for people to reduce their exposure to air pollution, for example in response to recommendations regarding the “most” and “least” favorable hours for staying outdoors. When there are high concentrations of air pollutants in the air, it is advisable to stay indoors. This is due to the inertia of the air in buildings, which can cause a “shift” and “lowering” of the daily maximum concentrations of several pollutants (Bouhambra et al. 2000; Hayes 1991; Chaloulakou and Mavroidis 2002). The flow balance of air pollution inside a building depends not only on outdoor air quality but also on the structure and air-tightness of the building, as well as indoor sources of pollution (Cichowicz et al. 2016; Cichowicz and Stelęgowski 2018b; Colbeck et al. 2008; De Paoli et al. 2018). However, according to the literature, there may be shifts of approximately 1 h and reductions of approximately 60% for CO (Chaloulakou and Mavroidis 2002), of 90% for O_3_ (Hayes 1991) and approximately 30% for benzene (Bouhambra et al. 2000). When there are low concentrations of pollutants outdoors, the situation is reversed. During such periods, it is better to stay outside, where concentrations can be half those in buildings (Chaloulakou and Mavroidis 2002).

## Automatic Measuring Stations in Cities, Towns, and Villages

Hourly measurements of pollutant concentrations in the ambient air are taken across a network of national air monitoring stations in Europe (Guerreiro et al. 2010; EEA 2016). These measurement stations are located in the vicinity of point, areal, and linear emission sources and provide data on the air quality in areas of, inter alia, “urban traffic/kerbside” (UT), “urban background” (CB), “suburban background” (SB), “rural background” (RB), and “natural background” (NB) pollution (Putaud et al. 2004).

There are various classifications of settlement units in European Union countries. To unify this division, the Organization for Economic Cooperation and Development with European Commission (OECD-EC) introduced the definition of a city as an urbanized area with a population of more than 50,000 and population density above 1500 inhabitants km^−2^ (Dijkstra and Poelman 2012). According to the definition developed in the European Territorial Observatory Network program (ESPON), towns are urbanized areas with populations of 5000–50,000 and population densities of 300–1500 inhabitants km^−2^ (Servillo et al. 2014).

## Daily Variations in Air Pollution

Daily variations in air-pollution level depend, to a large extent, on both meteorological conditions (Pérez et al. 2010) and human activity. Human activity profiles are relatively stable in particular areas, resulting in distinctive patterns air pollutant emissions (Gaffron 2012; Menut et al. 2012). However, the concentrations of air pollutants in a given area are influenced by many factors, none of which often is decisive. Depending on the location of the measuring station, the levels of air pollution can be more or less closely associated with particular factors. This has been confirmed by studies in which, for example, an analysis of the urban background pollution in the cities Canoas and Esteio in Brazil showed a correlation between O_3_ and solar radiation intensity, and no correlation between PM_10_ and air temperature (Agudelo-Castaneda and Teixeira 2014, 2017; Vouitsis et al. 2015). The Pearson correlation coefficient between O_3_ and radiation intensity was 0.558 (average) and − 0.186 (low) for PM_10_ and ambient temperature (Agudelo-Castaneda and Teixeira 2014). However, at a short distance from the emission source, the relationship was much stronger. This was confirmed by analyses carried out near a busy road in the city of Thessaloniki (Greece). The Pearson correlation coefficient between NO and CO and road traffic intensity was significantly stronger: 0.91 and 0.94 respectively in summer, and 0.89 and 0.40 in winter (Vouitsis et al. 2015). The changes in NO concentration during winter and summer seem related mainly to changes in road traffic intensity. Changes in CO concentration in winter were possibly associated with other factors, such as emissions from fuel combustion for heating purposes.

## Characteristics of the Tested Area

Poland is located in a temperate transitional climate zone. There are low temperatures in winter, reaching approximately − 14 °C (5th percentile) and on average ranging from approximately − 3 to + 1 °C (http://old.imgw.pl/klimat), depending on the region. As a result, there are high levels of pollution from fuel combustion for heating purposes (nonindustrial combustion). In contrast, temperatures in summer are significantly higher, reaching approximately + 28 °C (95th percentile), and on average ranging from approximately + 13 to + 19 °C (http://old.imgw.pl/klimat), depending on the region. Therefore, there are high levels of pollution from transport in summer. Most of Poland is located in the North European Plain, consisting of low plains. The north part of Poland is a coastal lowland adjacent to Baltic Sea, and the south part includes highlands and the Carpathian Mountains. The analyzed regions of Great Poland, Lodz, and Masovia are located in the lowlands (elevation up to 300 m). The Lublin region includes mostly lowlands, highlands (elevation up to 390 m), and Lower Silesia consists of lowlands, highlands (up to 718 m), and mountains (up to 1603 m). The chosen areas are similar for weather conditions. The annual average air temperature is 8 ± 1 °C, precipitation is approximately 600 mm, with higher values (up to 1300 mm) in the mountains (SP 2018). However, the prevailing rainfall occurs in summer. The mean monthly humidity usually range from 60 to 90%. The dominant wind direction is west, and the average velocity is 3 m s^−1^. The average insolation is 1600 h, and average solar radiation is 1000 kWh/m^2^. In 2015, approximately 91% of energy was generated from nonrenewable sources in Poland, primarily from hard coal and lignite (51%), crude oil (25%), and natural gas (15%) (IEA 2017). The share of urban population in the total population of a region amounts to approximately 46% in Lublin voivodeship, 55% in Greater Poland, 64% in Lodz and Masovia, and 69% in Lower Silesia. The dominant air contamination sources in the chosen areas are power industry, industrial and nonindustrial combustion, and road transport. Those economy sectors contribute to 97% of SO_2_, 80% of NO_x_, and 70% of PM_10_ emissions in Poland (Dębski et al. 2015; SP 2017). The amount of total gaseous emissions form point sources in 2017 was 12.0 × 10^6^ Mg/year in Lower Silesia, 14.4 × 10^6^ Mg/year for Greater Poland, 43.2 × 10^6^ Mg/year for Lodz, 29.1 × 10^6^ Mg/year for Masovia, and 5.0 × 10^6^ Mg/year for Lublin region. Dust emissions were 1.9 × 10^3^ Mg/year, 4.0 × 10^3^ Mg/year, 2.3 × 10^3^ Mg/year, 2.7 × 10^3^ Mg/year, and 1.7 × 10^3^ Mg/year respectively (https://bdl.stat.gov.pl). High point emissions in Lodz region were mainly caused by power industry, especially by large power plant “PGE GiEK” Belchatow. This power plant has 5342 MW_e_ electric power and uses brown coal as a fuel (Cichowicz and Stelegowski 2018c). It is placed 35 km from the analyzed location in Piotrków Trybunalski city, 60 km Lodz city, and 100 km from Gajew village. In Greater Poland region, there is the “ZE PAK” (http://zepak.pl/en) power plant complex (2512 × MW_e_, fuel: brown coal), placed 10–30 km from the analyzed locations in Konin city, 35 km from Piaski village, and 130 km from Poznan city. Also, each city and town in this analysis has its own, but smaller (< 500 MW_e_), power or combined heat and power plant, providing energy and/or heat for central part of its urbanized area.

Due to the specific characteristics of emissions in different areas, it was decided to analyze the changes in the average hourly concentrations of air pollutants at various locations in Poland, depending on the size of the settlement unit. The analysis focused on the selected air pollutants, which are harmful to human health: the gaseous substances NO_2_, NO_x_ (NO + NO_2_), SO_2_, O_3_ and CO, as well as the dusts PM_10_ and PM_2.5_ and benzene (C_6_H_6_). The analysis covered a period of 5 years from January 2012 to December 2016. The data were gathered from automatic air monitoring stations and were made available by the Voivodship Inspectorates for Environmental Protection (WIOŚ) and by the Main Inspectorate for Environmental Protection (GIOŚ) in Poland (http://powietrze.gios.gov.pl/pjp/current). The monitoring stations were categorized based on the character of the emissions, as city background (CB), town background (TB), or rural background (RB). Data from 15 monitoring stations were selected for analysis: five stations located in cities (CB stations), five in towns (TB stations), and five in villages (RB stations). The CB and TB stations were located in the surroundings of dense residential and service buildings, away from large point air pollution sources. RB measuring stations were located in the agriculture areas.

**Fig. 1:**
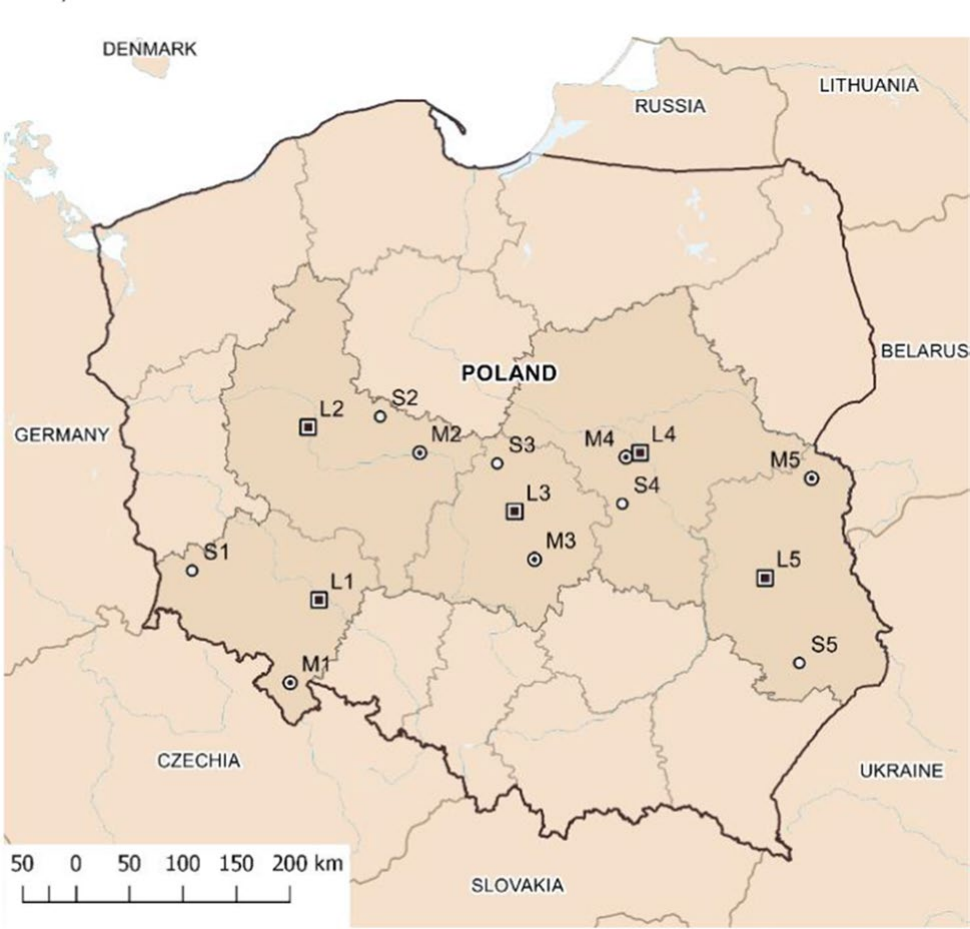
Location of measuring stations in selected cities, towns, and villages in Poland

Due to the prevailing western and south-western wind directions in Poland, all the selected measuring stations were on the “west–east” line, between 50 and 53°N latitude and 15–24°E longitude. This area comprised five provinces in central Poland (Fig. 1). In the following analysis, the locations are designated using symbols relating to the size of the settlement units and their location in a particular voivodship: L—city, M—town, S—village; 1—Lower Silesia voivodship, 2—Greater Poland voivodship, 3—Lodz voivodship, 4—Masovian voivodeship, 5—Lublin voivodeship (Tables 1, 2, 3).

**Table 1:** Location of CB measurement stations in cities

Symbol	City name	Population (inhabitants)	Density (inhabitants km^−2^)	Station address	City size
L1	Wrocław	639,000	2181	Korzeniowskiego St.	XL
L2	Poznań	539,000	2057	Polanka St.	XL
L3	Łódź	690,000	2354	Gdańska St.	XL
L4	Warsaw	1,765,000	3412	Marszałkowska St.	XXL
L5	Lublin	340,000	2310	Obywatelska St.	L

Population status as of 31 Dec 2017. City size is defined according to the nomenclature of OECD-EC classification, depending on the population of urban city center, as: L—large city, XL—extra-large city, XXL—extra extra-large city

**Table 2:** Location of TB measurement stations in towns

Symbol	Town name	Population (inhabitants)	Density (inhabitants km^−2^)	Station address	Town size
M1	Kłodzko	27,000	1098	Szkolna St.	Medium SMST
M2	Konin	75,000	919	Wyszyńskiego St.	Large SMST
M3	Piotrków Trybunalski	74,000	1114	Krakowskie Przedmieście St.	Large SMST
M4	Piastów	23,000	3977	Pułaskiego St.	Small SMST
M5	Biała Podlaska	57,000	1159	Orzechowa St.	Large SMST

Population status as of 31 Dec 2017. City size is defined according to the OECD-EC classification

**Table 3:** Location of RB measurement stations in villages

Symbol	Village name	Population (inhabitants)	Latitude	Longitude
S1	Osieczów	345	51°19′03.5″ N	15°25′54.2″ E
S2	Piaski	124	52°30′05.0″ N	17°46′24.5″ E
S3	Gajew	114	52°08′35.7″ N	19°41′49.0″ E
S4	Belsk Duży	790	51°50′06.4″ N	20°47′29.6″ E
S5	Obrocz	525	50°35′26.9″ N	22°59′52.4″ E

Population status as of 31 Dec 2009

The measurements were made at automatic air monitoring stations, according to the reference or equivalent methods (Table 4). However, due to technical/service breaks in the operation of individual measuring stations, full data could not be obtained for all gaseous substances and dusts. Therefore, only the data from years with at least 75% completeness for a particular station were used in the analysis. The average hourly concentrations were thus determined based on the number of years that met this criterion for a given station (Table 5).

**Table 4:** Methods of measuring air pollution at measuring stations

Pollutant	Measuring method
NO, NO_2_	Chemiluminescence, according to EN 14211; precision ± 0.5%
SO_2_	Ultraviolet fluorescence, according to EN 14212; precision ± 1%
CO	Infrared spectroscopy, according to EN 14626; precision ± 0.1 ppm
O_3_	Ultraviolet photometry, according to EN 14625; precision ± 1 ppb
PM_10_, PM_2.5_	Beta attenuation, according to EN 12341; precision ± 2 µg m^−3^
benzene	Gas chromatography with flame-Ionization detection, according to EN 16450:2017-05; precision ± 3.0%

**Table 5:** Number of measuring stations and the number of years that met the criterion of completeness for results

Pollutant	Stations × years		
	CB stations	TB stations	RB stations
NO_2_	25	22	25
NO_x_	25	20	25
O_3_	14	23	25
SO_2_	20	24	25
CO	23	10	5
PM_10_	18	15	7
PM_2.5_	12	2	n/a
benzene	12	n/a	n/a

In Poland, the levels and nature of air pollutants vary significantly between in winter periods (WPs) and summer periods (SPs). Therefore, the measurements were divided into two seasons. It was assumed that WP lasted from October 1 to March 31 and covered the entire heating season, whereas SP lasted from May 1 to August 31 and covered the vegetation period.

## Results and Discussion

The analysis of average hourly pollutant concentrations in the ambient air at stations CB, TB, and RB, in WP and SP in the years 2012–2016, revealed daily variations in air pollution levels. The periods during a “typical” day were identified when the daily maximum and daily minimum average hourly concentrations of pollutants occurred.

**Fig. 2:**
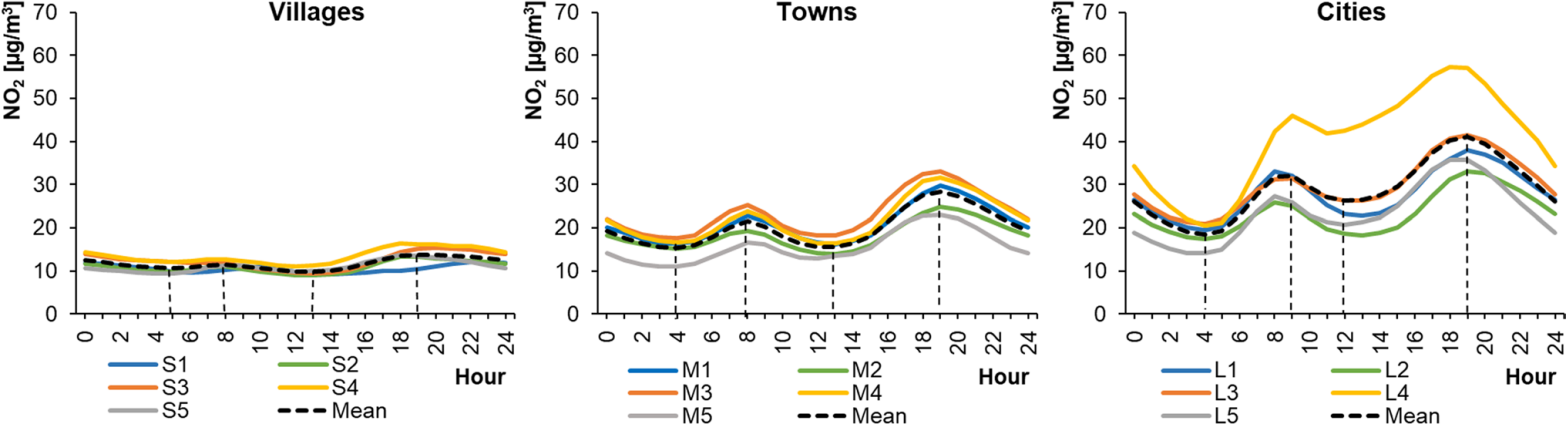
Average hourly NO_2_ concentrations in winter periods

**Fig. 3:**
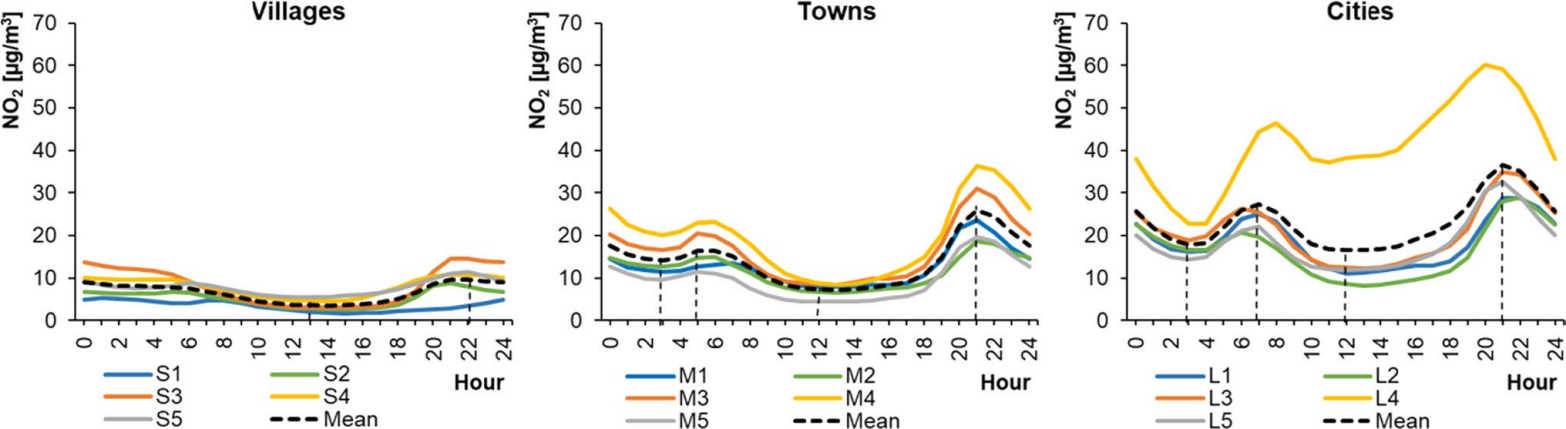
Average hourly NO_2_ concentrations in summer periods

Mean hourly NO_2_ concentrations in WPs ranged from 14.12 to 57.27 μg m^−3^ in cities, from 11.06 to 32.98 μg m^−3^ in towns, and from 9.10 to 16.41 μg m^−3^ in rural areas (Fig. 2). In SPs, the mean hourly NO_2_ concentrations ranged from 8.29 to 60.10 μg m^−3^, from 4.47 to 36.33 μg m^−3^, and from 1.75 to 4.47 μg m^−3^ for cities, towns, and rural areas, respectively (Fig. 3). This means that the lowest concentrations occurred during SPs and the highest in winter, except at locations L4 and M4 (stations in the Warsaw city and the town of Piastów in the Mazovian Province). The shape of the NO_2_ daily profile was always close to a bimodal distribution, with two distinct minima and maxima. The daily maximum concentration was higher in the evenings than in the morning. The lowest concentrations occurred in SPs in the afternoon and, importantly, remained at a similar level for 6–12 h. However, these periods of low NO_2_ concentrations were much shorter, lasting 1–4 h. The shapes of the hourly profiles for the same type of settlement unit were similar.

**Fig. 4:**
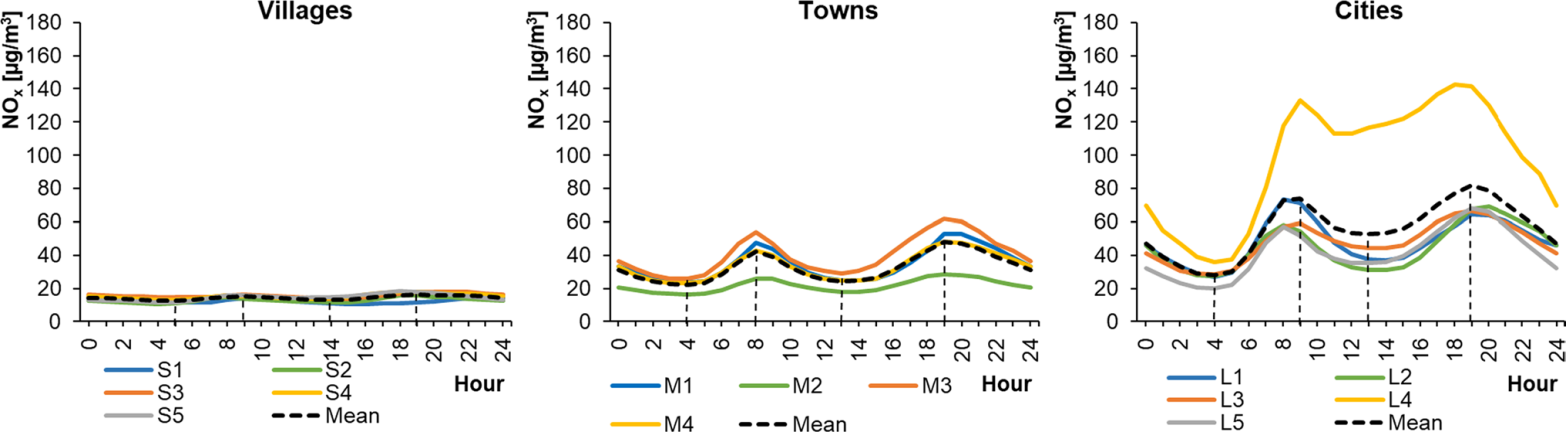
Average hourly NO_x_ concentrations in winter periods

**Fig. 5:**
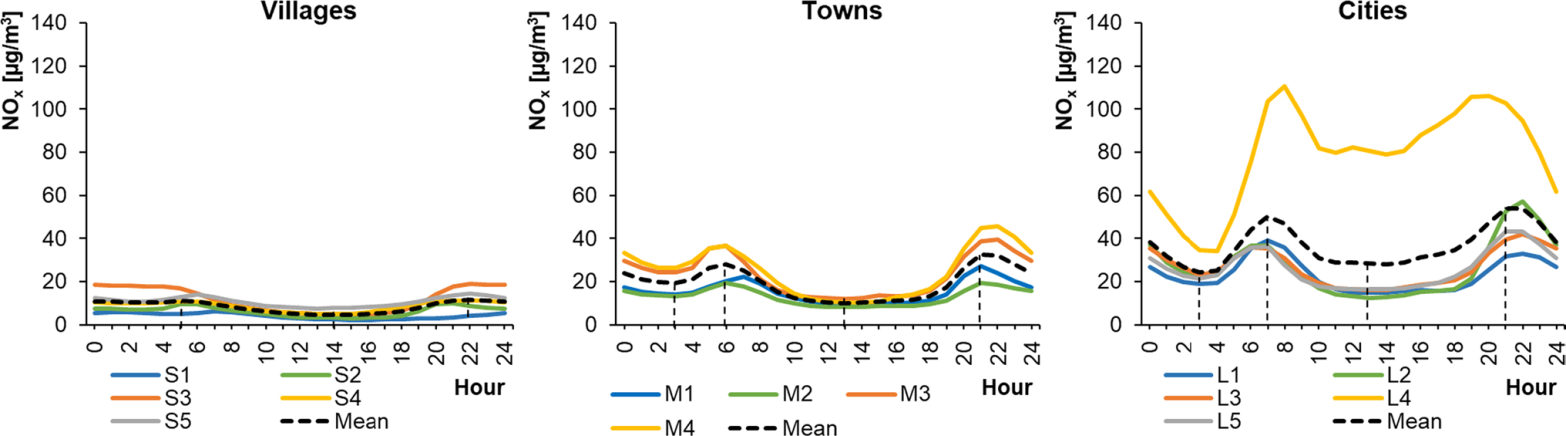
Average hourly NO_x_ concentrations in summer periods

The average hourly NO_x_ concentrations in WPs ranged from 20.27 to 142.77 μg m^−3^ in cities, from 16.54 to 61.86 μg m^−3^ in towns, and from 10.74 to 18.33 μg m^−3^ in rural areas (Fig. 4). The concentrations were lower in SPs and ranged from 12.56 to 110.57 μg m^−3^, from 8.11 to 45.79 μg m^−3^, and from 2.16 to 18.80 μg m^−3^, respectively (Fig. 5). The shape of the NO_x_ daily profiles was close to a bimodal distribution, similar to the NO_2_ profiles. However, in the case of NO_x_ the morning and evening peaks had similar values. This was most likely caused by NO emissions resulting from the morning rush hours. However, in summer in the cities of Wrocław (L1) and Warsaw (L4), the morning NO_x_ peak concentrations were higher than those in the evening, probably due to the heavier traffic congestion. The shapes of the NO_x_ profiles were similar for particular types of settlement unit. The NO_x_ level was also lower during SPs than in WPs in the cities and towns. Similar results were previously reported (Schneider et al. 2016; Garcia et al. 2014; Martinello et al. 2014).

**Fig. 6:**
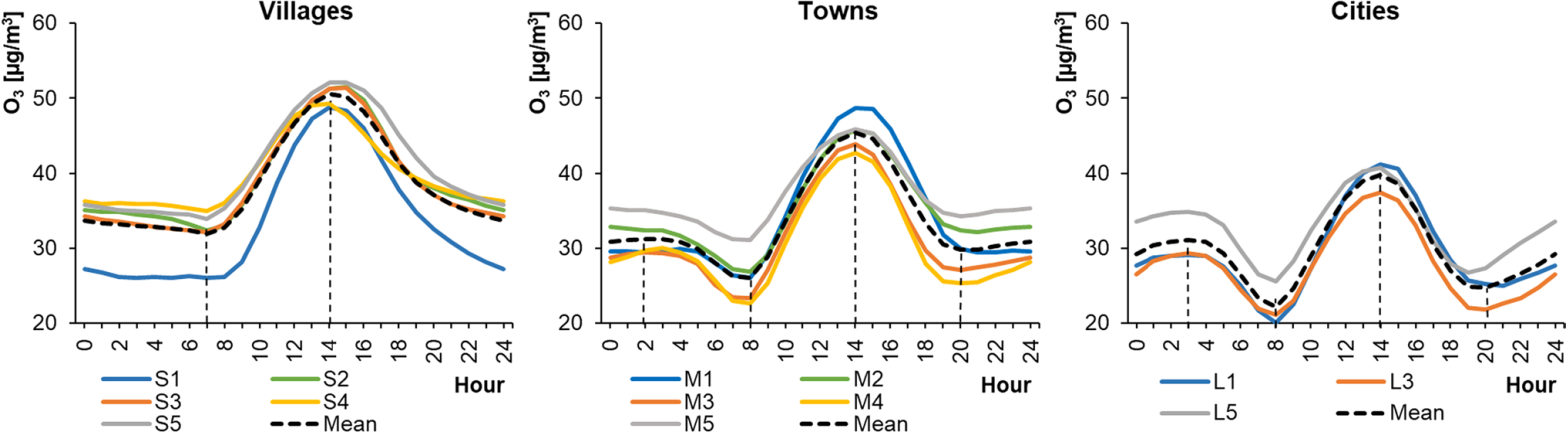
Average hourly O_3_ concentrations in winter periods

**Fig. 7:**
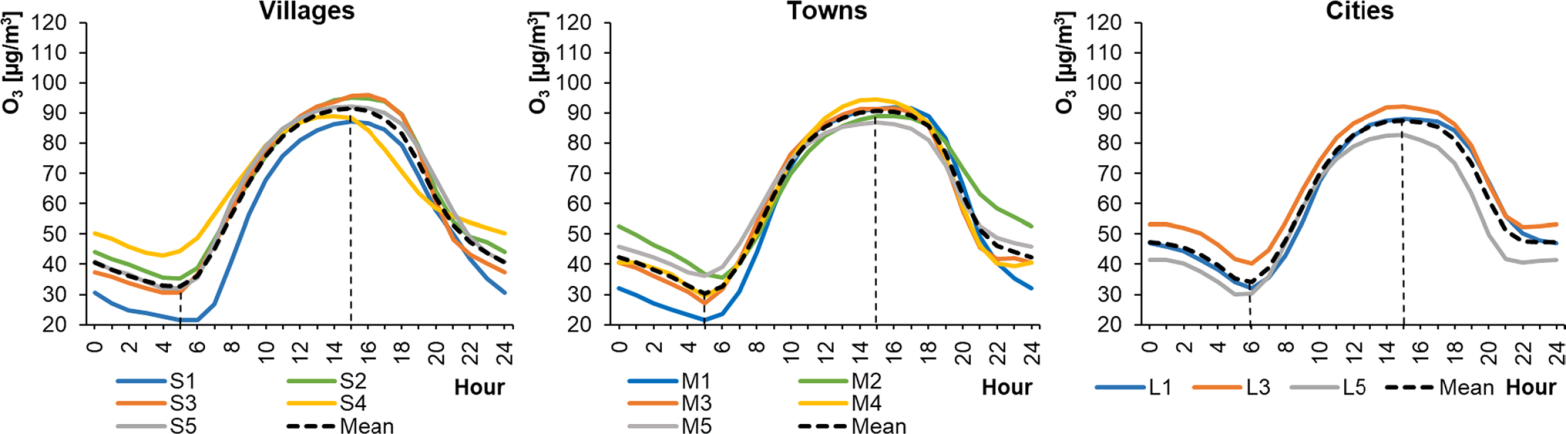
Average hourly O_3_ concentrations in summer periods

The average hourly O_3_ concentrations in WPs ranged from 20.02 to 41.23 μg m^−3^ in cities, from 22.62 to 48.73 μg m^−3^ in towns, and from 25.98 to 52.10 μg m^−3^ in rural areas (Fig. 6). The concentrations were higher in SPs, ranging from 29.98 to 92.27 μg m^−3^ in cities, from 21.61 to 94.67 μg m^−3^ in towns, and from 21.46 to 96.08 μg m^−3^ in rural areas (Fig. 7). Ground-level ozone concentrations generally increased as the size of the settlement unit decreased. This confirms the results of previous scientific studies (Guerreiro et al. 2010; Hagenbjörk et al. 2017), which showed that the level of O_3_ decreases as the NO concentration in the air rises. The NO level was usually higher in those areas with heavier traffic. However, the lowest O_3_ concentrations in SPs occurred in cities and were higher than the minimal values in towns and villages. Daily fluctuations in O_3_ levels were the widest in rural areas. This contradicts research into ground-level ozone concentrations in different types of area carried out by Zheng et al. (2010), who reported smaller ozone changes in villages than in cities. The shapes of the ozone profiles were similar for particular types of settlement unit. The daily profiles for O_3_ concentrations were similar to a bimodal distribution in the case of towns and cities, and unimodal for the villages. The maximum values occurred, irrespective of the season and the size of the city/village, at around 14:00–15:00 local time. The sudden drop in O_3_ concentration was more significant in the case of larger settlement units and occurred at 7:00–8:00 (local time) in WPs and around 5:00 (local time) in SPs. This could be related to the decrease in the level of ozone, which reacted with nitrogen oxide emitted during the morning rush hours. The shape of the O_3_ diurnal profile in cities during winter is puzzling, because the level of ozone at night increased and at around 3:00 (local time) reached almost ¾ of the maximum daily value.

**Fig. 8:**
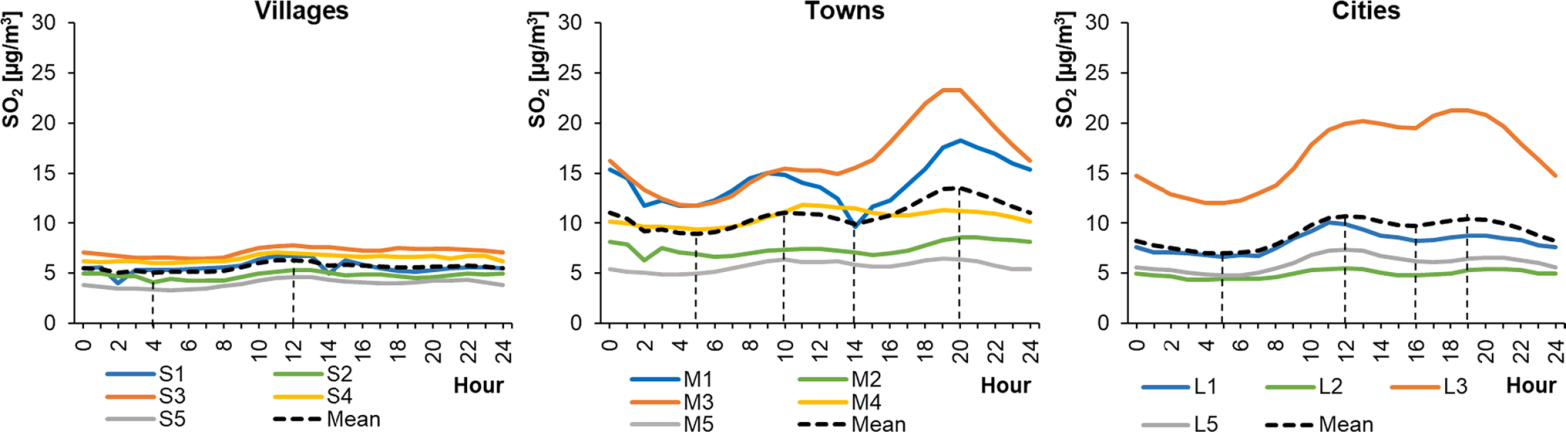
Average hourly SO_2_ concentrations in winter periods

**Fig. 9:**
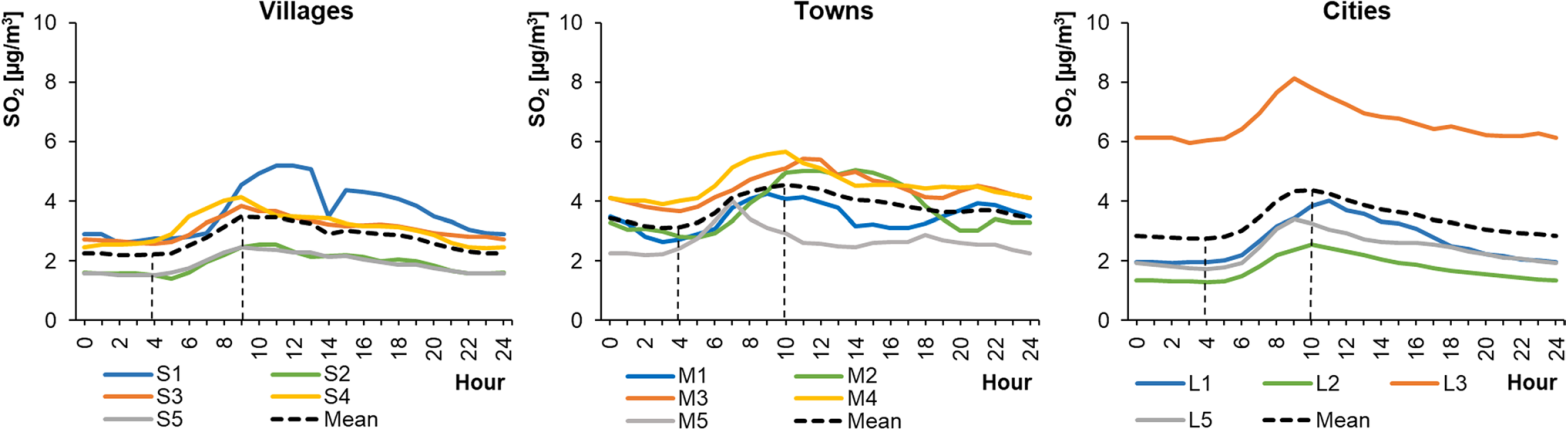
Average hourly SO_2_ concentrations in summer periods

The average hourly SO_2_ concentrations in WPs ranged from 4.36 to 21.27 μg m^−3^ in cities, from 4.84 to 23.29 μg m^−3^ in towns, and from 3.28 to 7.74 μg m^−3^ in rural areas (Fig. 8). The concentrations were much lower in SPs, ranging from 1.30 to 8.12 μg m^−3^ in cities, from 2.11 to 5.65 μg m^−3^ in towns, and from 1.40 to 5.19 μg m^−3^ in rural areas (Fig. 9). The level of SO_2_ was generally the lowest in villages and higher in towns and cities. In the Greater Poland and Lublin regions, the concentrations of sulphur dioxide in the cities of Poznań (L2) and Lublin (L5) were lower and comparable to those occurring in the towns of Piaski (S2) and Biały Słup (S5). The highest SO_2_ level occurred in the Łódź region (Gajew/S3, Piotrków Trybunalski/M3, and in Łódź/L3), where there is a very extensive network of power stations. Therefore, it was assumed that changes in SO_2_ concentrations were related to industrial power production in SPs and to emissions from the combustion of fuels for heating individual buildings in WPs. The shapes of the SO_2_ daily profiles were similar to unimodal (in SPs) or bimodal (in WPs) distributions. The unimodal character of the profiles was more pronounced in SPs than in WPs, with a peak around 10:00 local time.

**Fig. 10:**
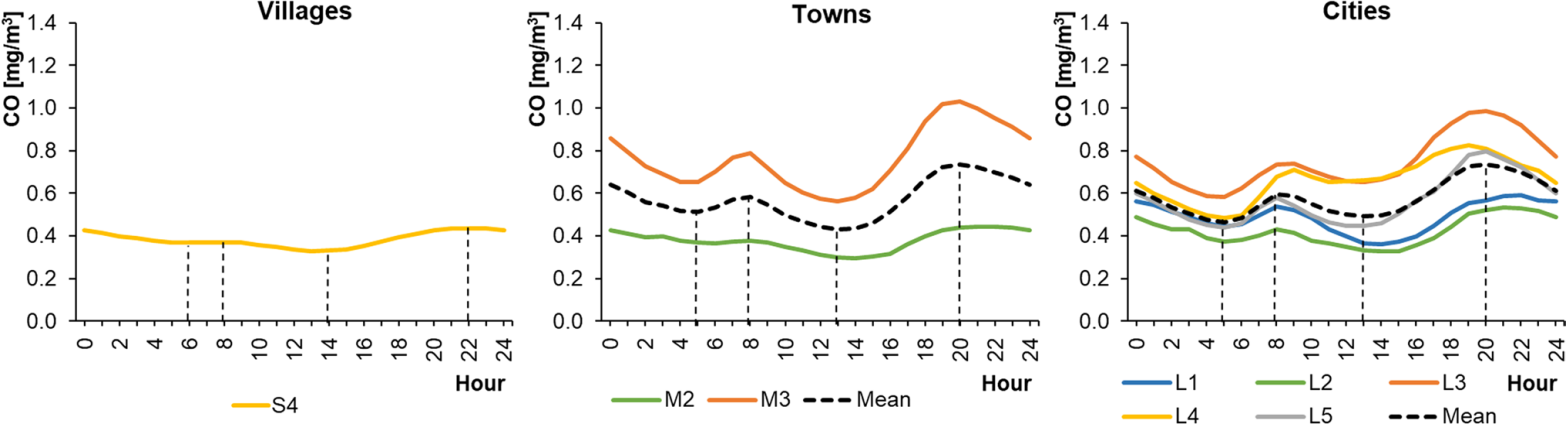
Average hourly CO concentrations in winter periods

**Fig. 11:**
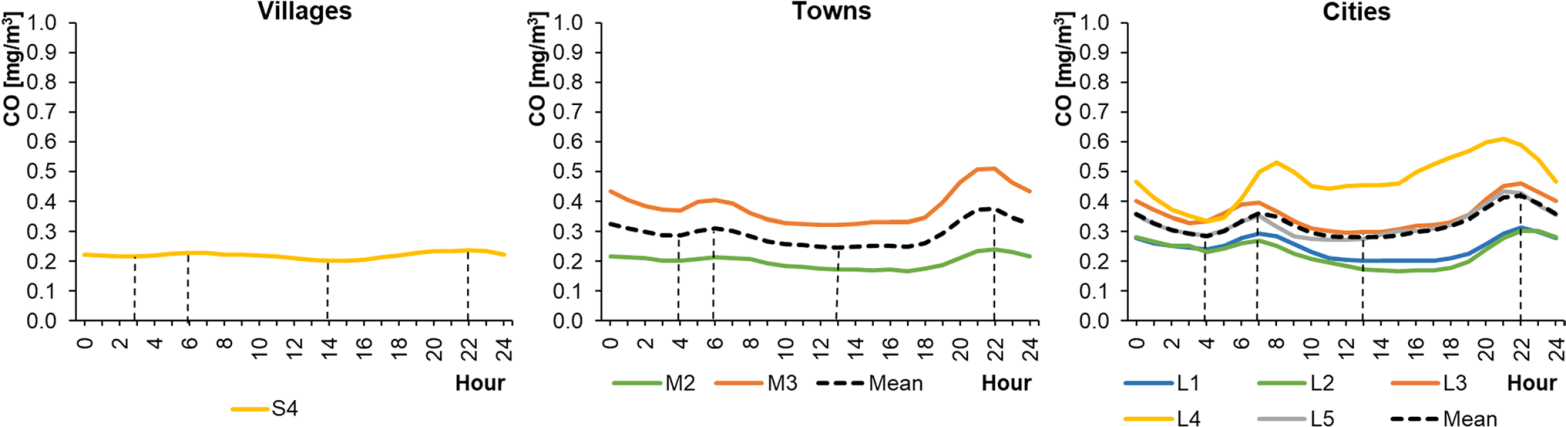
Average hourly CO concentrations in summer periods

Of the 15 selected monitoring stations, 8 measured CO concentrations in the ambient air. These were located mainly in cities. Unfortunately, there were no CO measuring devices installed at the other 7 stations, located in smaller settlement units. The average hourly CO concentrations in WPs ranged from 0.33 to 0.99 mg m^−3^ in cities, from 0.29 to 1.03 mg m^−3^ in towns, and from 0.33 to 0.44 mg m^−3^ in rural areas (Fig. 10). The concentrations were lower in SPs, ranging from 0.17 to 0.61 mg m^−3^ in cities, from 0.17 to 0.51 mg m^−3^ in towns, and from 0.20 to 0.24 mg m^−3^ in rural areas (Fig. 11). The CO level in the countryside changed only slightly during the day, in contrast to the fluctuations observed in towns and cities. The CO profiles were similar to a bimodal distribution and comparable to the NO_2_ daily profiles. The highest peak in CO concentrations was in the evening, at 20:00–22:00 (local time), in both WP and SP.

**Fig. 12:**
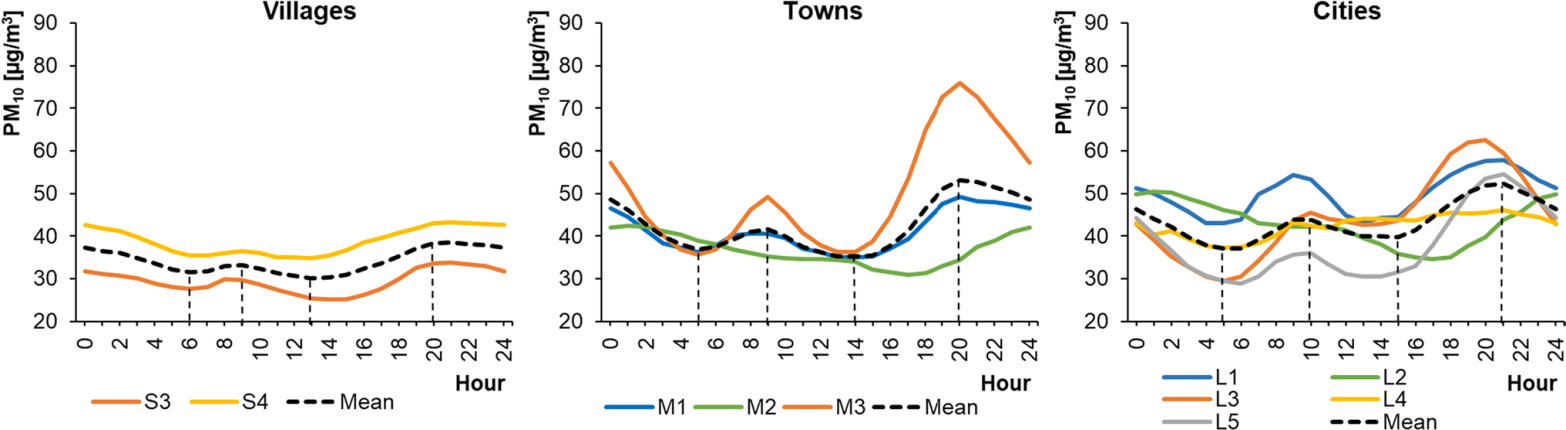
Average hourly PM_10_ concentrations in winter periods

**Fig. 13:**
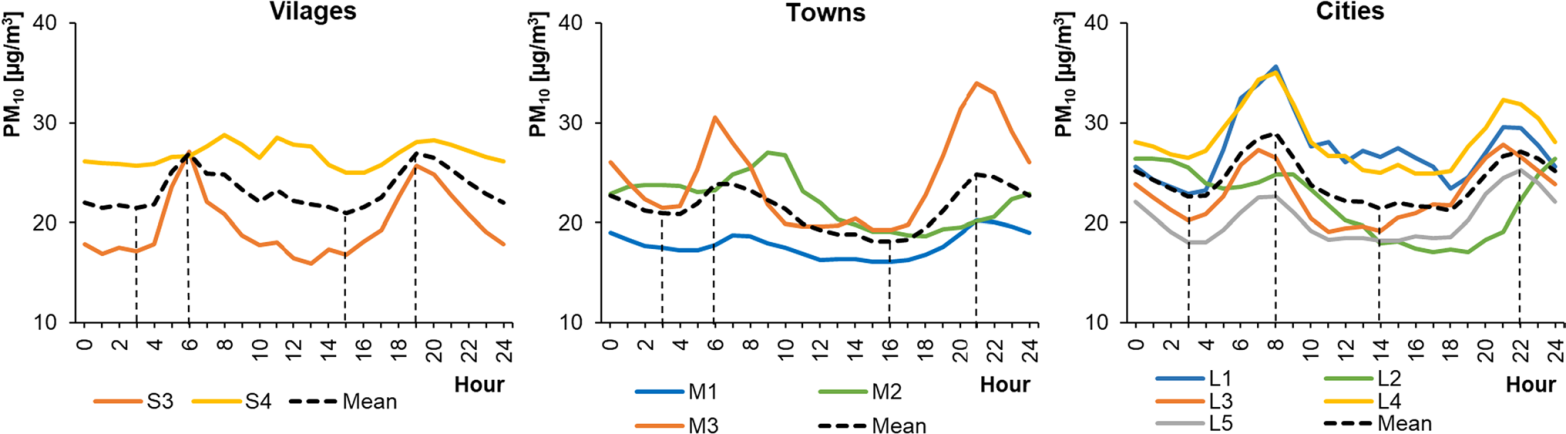
Average hourly PM_10_ concentrations in summer periods

Of the 15 selected monitoring stations, 10 measured PM_10_ concentrations in the ambient air. These were mainly located in cities. Unfortunately, there were no PM_10_ measuring devices installed at the other 5 stations, located in smaller settlement units. The average hourly PM_10_ concentrations in WPs ranged from 28.76 to 62.54 μg m^−3^ in cities, from 31.00 to 75.87 μg m^−3^ in towns, and from 25.07 to 43.16 μg m^−3^ in rural areas (Fig. 12). The concentrations in SPs ranged from 17.08 to 35.63 μg m^−3^ in cities, from 16.09 to 34.00 μg m^−3^ in towns, and from 15.90 to 28.78 μg m^−3^ in rural areas (Fig. 13). Therefore, during WPs the PM_10_ levels in towns and cities were similar. In villages, they were approximately 50% lower. The PM_10_ profiles at each location were characterized by a bimodal distribution but with different shapes. The highest PM_10_ concentrations during WPs occurred in the evening. This was in contrast to during SPs, when some locations (Wrocław/L1, Warsaw/L4, Konin/M2, and Gajew/S3) saw their highest PM_10_ concentrations in the morning, around 6:00–8:00 (local time). In others (Poznań/L2, Łódź/L3, Lublin/L5, Kłodzko/M1, Piotrków Trybunalski/M3, and Belsk Duży/S4), the highest peak occurred in the evening at 21:00–22:00 (local time).

**Fig. 14:**
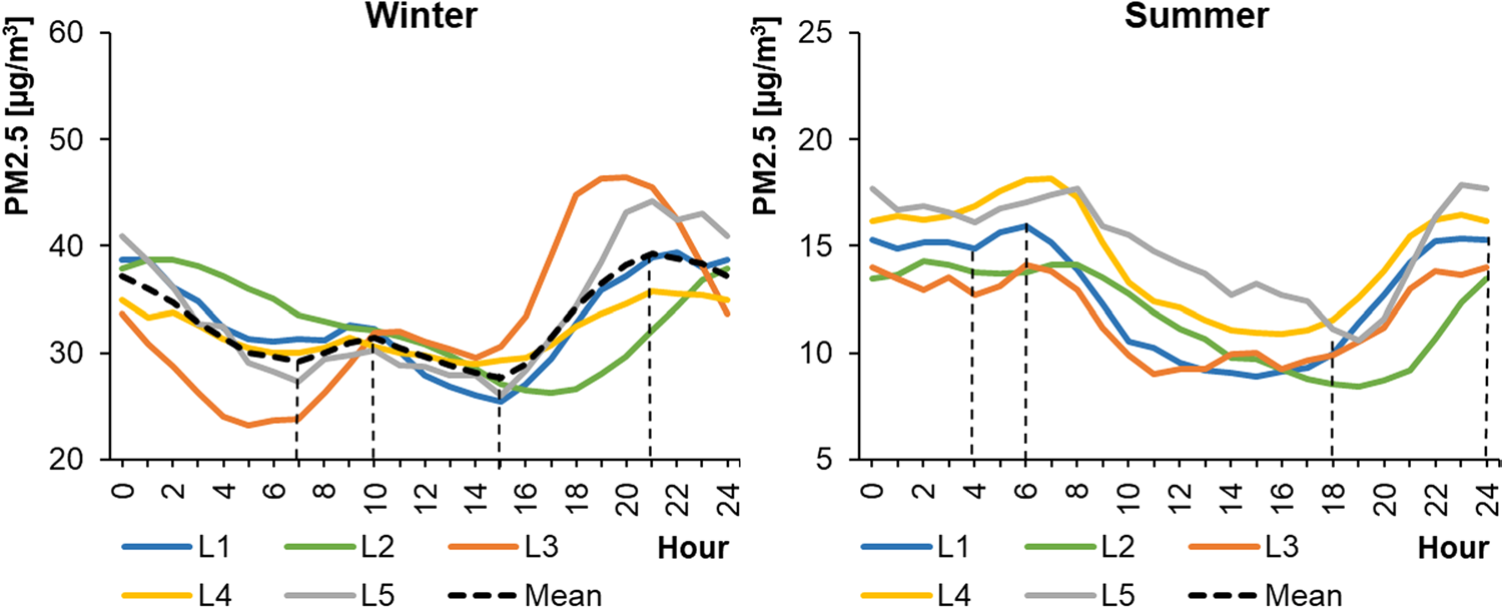
Average hourly PM_2.5_ concentrations in cities

In the Polish air quality monitoring system, only CB stations measure PM_2.5_ and benzene concentrations in the ambient air. The average hourly concentrations of PM_2.5_ in cities differed significantly depending on the season, ranging from 23.24 to 46.42 μg m^−3^ in WPs and from 8.40 to 18.18 μg m^−3^ in SPs (Fig. 14). The concentrations in SPs were approximately three times smaller than during WPs and were lowest from 10:00 to 18:00 local time (depending on the location). The maximum values during WPs occurred in the evening at approximately 21:00 (local time). In SPs, the peak hours occurred between 6:00 and 24:00 local time. Interestingly, there were high concentrations of PM_2.5_ throughout the night in SPs. Previous research on the composition of dust in urban areas of Barcelona, Spain (Pérez et al. 2010) had also indicated the presence of large amounts of organic matter, molecular carbon, and aerosols in PM_1–2.5_ (approximately 48% of mass) and in PM_1_ (around 85%) in urban ambient air. While in PM_2.5–10_ it contributed only to 24%. This is puzzling, because sources of anthropogenic emissions are inactive at night during summer months. Therefore, it can be assumed that the increase of PM_2.5_ concentrations at night might be associated with the condensation of water vapor and the coagulation of fine dust particles (Pérez et al. 2010). However, in the study of air quality in Thessaloniki, Greece (Vouitsis et al. 2015), the concentrations of particle number (PNC), PM_2.5_, and PM_10_, increased during the night (with peak at 23:00–24:00) mostly in the winter period in traffic-affected sites, whereas in city-background locations this was not clearly noticeable. On the other hand, in this analysis, PM_2.5_ concentration decreased at night in winter, until around 3:00 local time. The differences in the shapes of the profiles and peak hours for PM_10_ and PM_2.5_ also were interesting. The diurnal variations in PM_10_ concentration took various shapes in cities, whereas the concentrations of PM_2.5_ followed very similar patterns. In addition, PM_2.5_ concentrations in the morning peak occurred 2 h earlier than the PM_10_ peak hours. In the study by Pérez et al. (2010), at weekdays the peaks in PM1 and PM1–2.5 occurred at 6:00–9:00, whereas peak in PM_2.5–10_ occurred at 10:00–15:00. At weekends, it was around 2:00–8:00 and 11:00–12:00, respectively. However, in the case of traffic-affected site in the center of Madrid, Spain, the peaks in PM_2.5_ and PM_10_ were very close to each other (± 0.5 h) and occurred at 8:00–9:00 and 20:00–21:00 (Moreno et al. 2013). Also, in urban areas, the average PM_2.5_ concentration are usually strongly correlated (R^2^ > 0.80) to PM_10_ and amounted to 60–90% of PM_10_ concentrations (Gupta et al. 2006; Pérez et al. 2010). Nevertheless, observations of PM could indicate that differences in the varying concentrations of course and finer particles in the urban environment might be partially due to the different processes of their formation (Pérez et al. 2010).

**Fig. 15:**
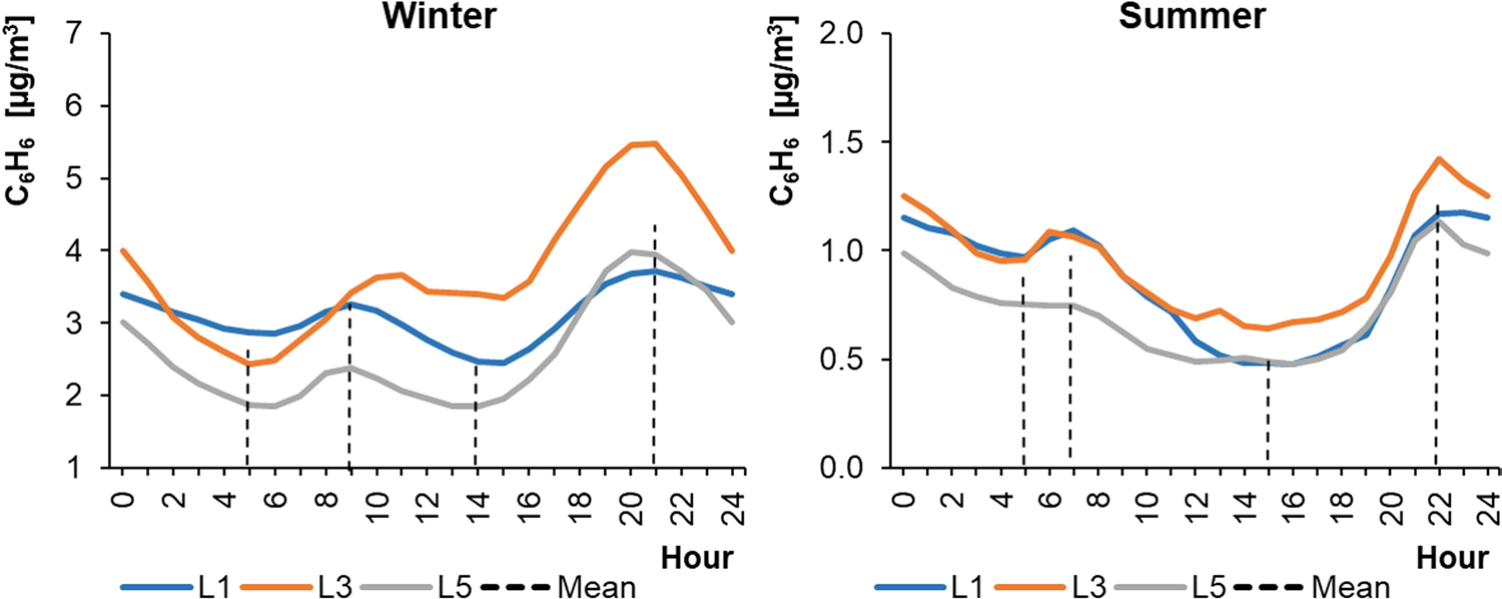
Average hourly concentrations of benzene in cities

As in the case of PM_2.5_, the concentrations of benzene (C_6_H_6_) in cities changed and varied significantly depending on the season. The average hourly benzene concentrations ranged from 0.48 to 1.42 μg m^−3^ in WPs and from 1.84 to 5.47 μg m^−3^ in SPs (Fig. 15). The benzene level in the summer was three to four times lower than in winter. The highest concentrations always occurred in the evening, between 21:00 and 22:00 local time.

**Fig. 16:**
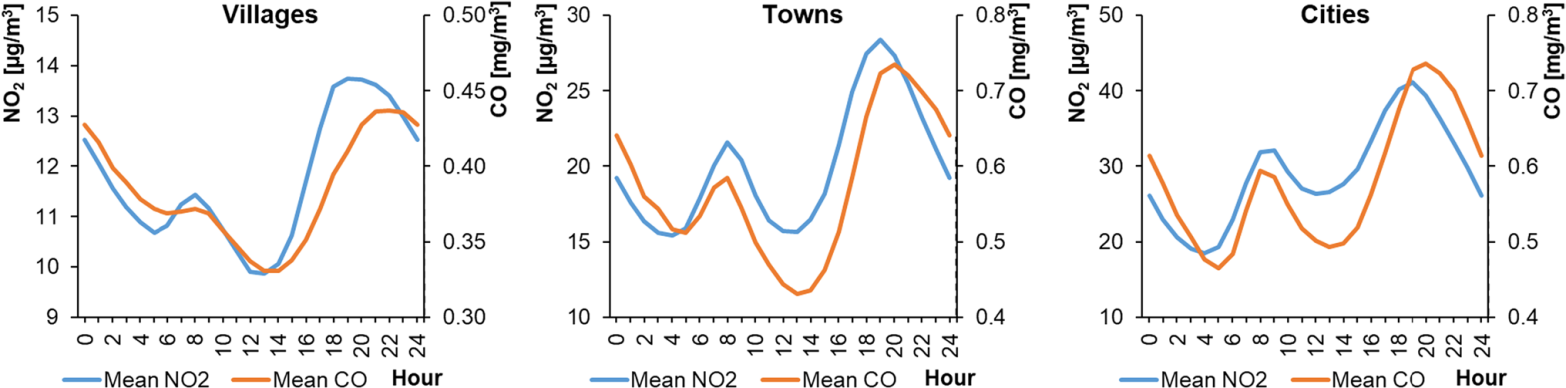
Comparison of average hourly NO_2_ and CO profiles in WPs

**Fig. 17:**
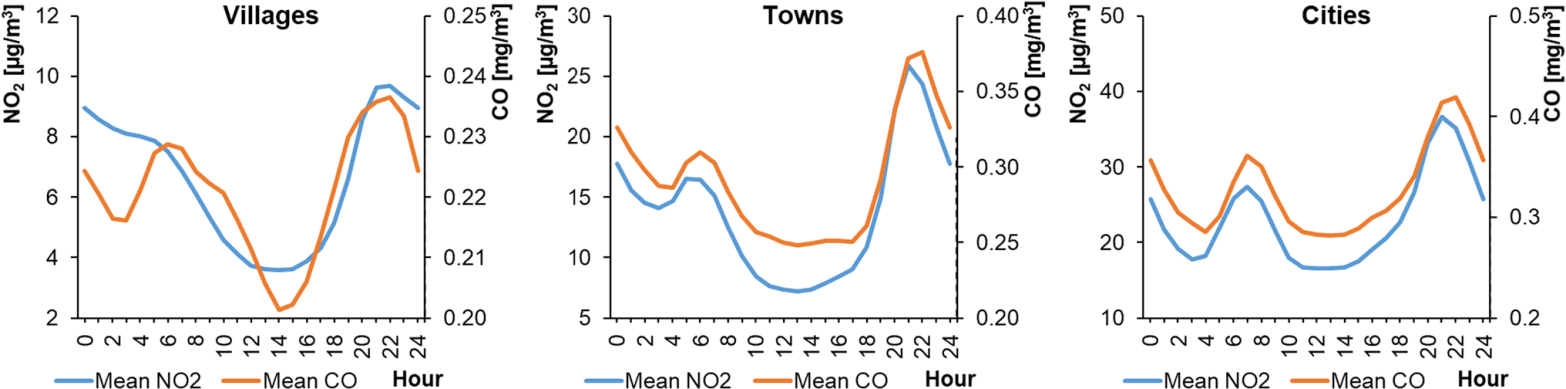
Comparison of average hourly NO_2_ and CO profiles in SPs

**Fig. 18:**
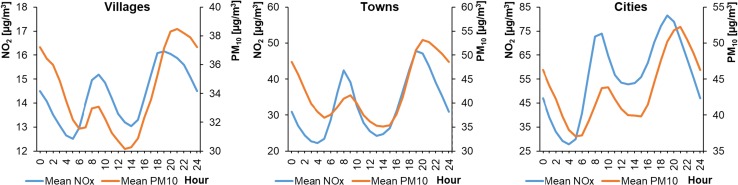
Comparison of average hourly NO_x_ and PM_10_ profiles in WPs

**Fig. 19:**
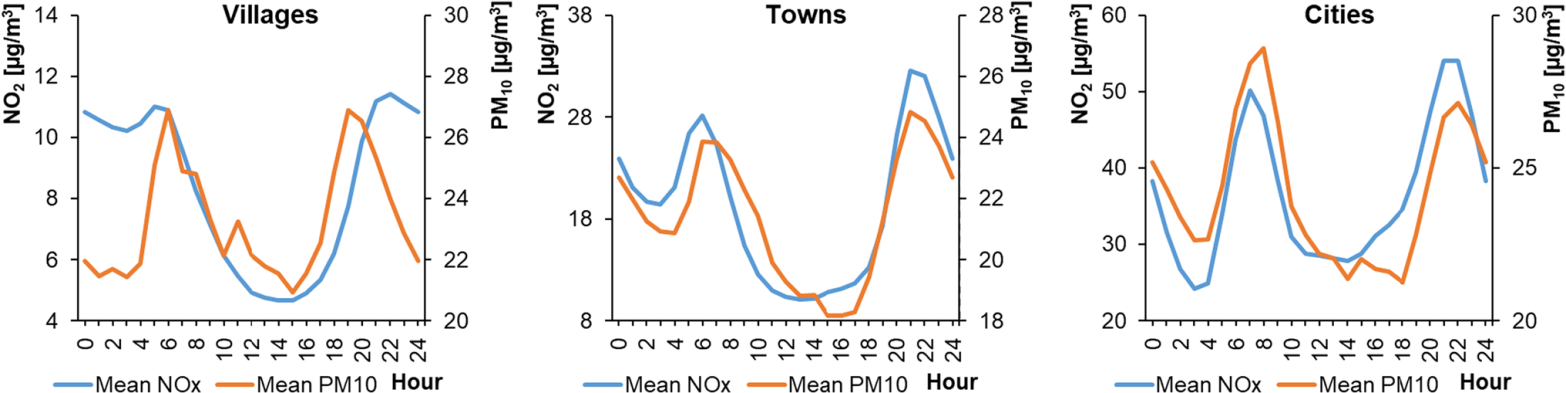
Comparison of average hourly NO_x_ and PM_10_ profiles in SPs

The shapes of the average daily profiles of NO_2_ and CO, and NO_x_ and PM_10_ were compared for different settlement units (Figs. 16, 17, 18, 19). This was to verify the generally prevailing opinion that the NO_2_ and NO_x_ concentrations usually indicate road transport intensity, whereas the occurrence of CO and PM_10_ indicate emissions from road transport and area sources (so-called “low emission”). It was observed that there was a co-occurrence of changes in the concentrations, as well as of “peak hours” and “low hours,” in both WPs and SPs. The high correspondence between the profile shapes in SPs could indicate the impact of emissions from linear sources. However, it is more difficult to explain their consistency in WPs, when there also were area emissions related to the heating of individual buildings.

**Fig. 20:**
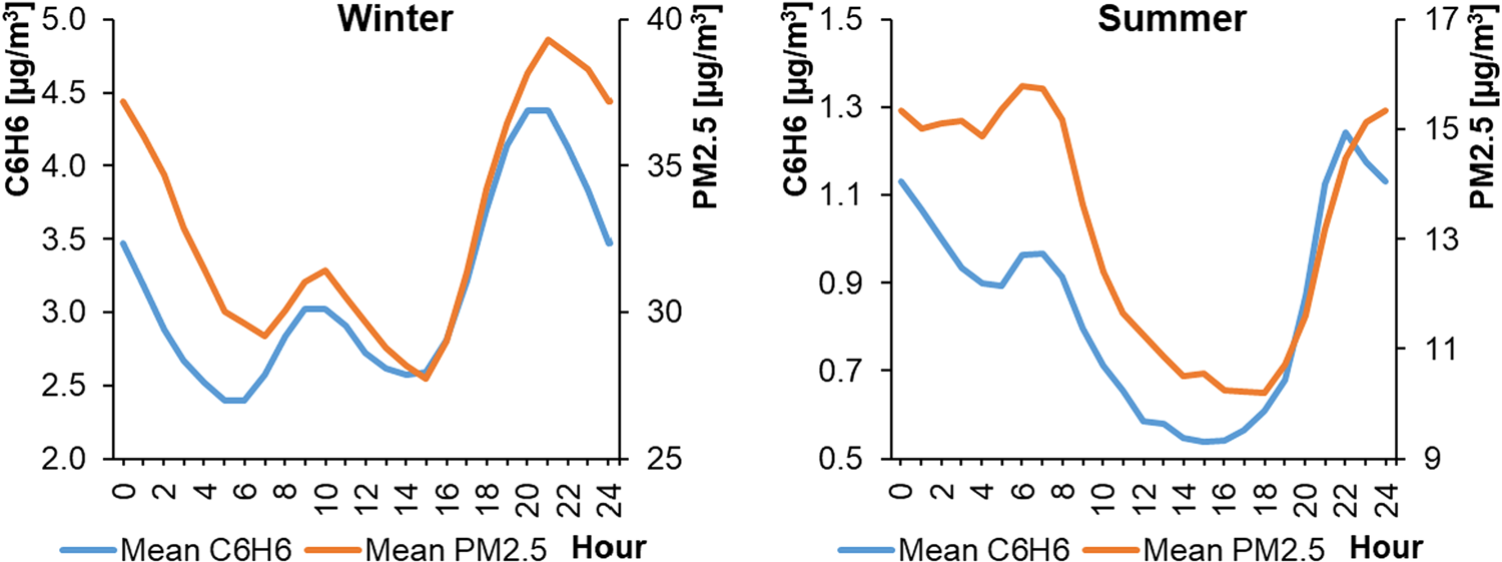
Comparison of average hourly benzene and PM_2.5_ profiles in cities

The shapes of the average hourly benzene and PM_2.5_ profiles were compared for cities during WPs and SPs (Fig. 20). The presence of PM_2.5_ in the ambient air is due mainly to the burning of fossil fuels (50% of energy in Poland is generated from hard coal and lignite, while crude oil provides 26% and natural gas 15% of the country’s energy needs). Approximately 76% of PM_2.5_ emissions originates from fuel combustion in power generation, industrial, and nonindustrial combustion (Dębski et al. 2015). The presence of benzene results from tobacco smoke and from burning liquid fuels in car engines (WHO 2000). Also, in the transport-affected sites, the carbonaceous compounds are substantial (35–48%) components of PM_2.5_ (Gómez-Perales et al. 2004; Harrison et al. 2004, Pérez et al. 2010). In the analyzed locations, the changes in PM_2.5_ and benzene levels had similar hourly profiles, indicating the co-occurrence of these air pollutants in urbanized areas. The co-existence of PM and benzene (correlation coefficient of 0.69–0.97) during the transport rush hours were previously reported (Gupta et al. 2006).

There were significant shifts between the occurrence of the daily maximum and daily minimum in WPs and SPs (Tables 6, 7, 8). The most favorable conditions, in terms of levels of air pollution, occurred in towns and cities in the morning at 3:00–5:00 (local time) and in the afternoon at 12:00–15:00. In the villages, the best air quality occurred between 4:00 and 6:00 (local time). On the other hand, during WPs the least favorable conditions in terms of air quality occurred, regardless of location at 8:00–10:00 and 19:00–21:00 (local hour). In SPs, air quality was poorest at the following times: 7:00–8:00 and 21:00–22:00 in cities; 5:00–6:00 and 21:00–22:00 in towns; 6:00–9:00 and 19:00–22:00 in villages. For a comparison, when considering PM and NO_2_ air contamination, in urban-traffic area in Madrid (Spain), the most favorable conditions occurred at around 4:00 and 15:00 local hour (Moreno et al. 2013). In Barcelona (Spain), this was around 15:00 (Pérez et al. 2010), and in Thessaloniki (Greece) around 4:00–6:00 and 15:00–18:00 (Vouitsis et al. 2015). For comparison, in the cities of Canoas and Esteio, in Brazil, the most favorable air quality conditions occurred at 4:00–6:00 and 14:00–17:00 (Agudelo-Castaneda and Teixeira 2014).

**Table 6:** Daily maximum and daily minimum average concentrations of air pollutants in cities (CB stations)

	Peak hours				Low hours			
	Winter period		Summer period		Winter period		Summer period	
	Morning	Evening	Morning	Evening	Morning	Evening	Morning	Evening
NO_2_	09:00	19:00	07:00	21:00	04:00	12:00	03:00	12:00
NO_x_	09:00	19:00	07:00	21:00	04:00	13:00	03:00	13:00
O_3_	03:00	14:00	n/a	15:00	08:00	20:00	06:00	n/a
SO_2_	12:00	19:00	10:00	n/a	05:00	16:00	04:00	n/a
CO	08:00	20:00	07:00	22:00	05:00	13:00	04:00	13:00
PM_10_	10:00	21:00	08:00	22:00	03:00	14:00	03:00	14:00
PM_2.5_	07:00	21:00	06:00	24:00	03:00	12:00	04:00	18:00
C_6_H_6_	09:00	21:00	07:00	22:00	05:00	14:00	05:00	15:00

**Table 7:** Daily maximum and daily minimum average concentrations of air pollutants in cities (TB stations)

	Peak hours				Low hours			
	Winter period		Summer period		Winter period		Summer period	
	Morning	Evening	Morning	Evening	Morning	Evening	Morning	Evening
NO_2_	08:00	19:00	05:00	21:00	04:00	13:00	03:00	12:00
NO_x_	08:00	19:00	06:00	21:00	04:00	13:00	03:00	13:00
O_3_	02:00	14:00	n/a	15:00	08:00	20:00	05:00	n/a
SO_2_	10:00	20:00	10:00	n/a	05:00	14:00	04:00	n/a
CO	08:00	20:00	06:00	22:00	05:00	13:00	04:00	13:00
PM_10_	09:00	20:00	06:00	21:00	05:00	14:00	03:00	16:00

**Table 8:** Daily maximum and daily minimum average concentrations of air pollutants in villages (RB stations)

	Peak hours				Low hours			
	Winter period		Summer period		Winter period		Summer period	
	Morning	Evening	Morning	Evening	Morning	Evening	Morning	Evening
NO_2_	08:00	19:00	n/a	22:00	05:00	13:00	n/a	13:00
NO_x_	09:00	19:00	07:00	21:00	04:00	13:00	03:00	13:00
O_3_	n/a	14:00	n/a	15:00	07:00	n/a	05:00	n/a
SO_2_	n/a	12:00	09:00	n/a	04:00	n/a	04:00	n/a
CO	08:00	22:00	06:00	22:00	06:00	14:00	03:00	14:00
PM_10_	09:00	20:00	06:00	19:00	06:00	13:00	03:00	15:00

According to the literature (Gaffron 2012; Menut et al. 2012; Moreno et al. 2013; Pérez et al. 2010; Vouitsis et al. 2015), daily variations in NO_2_ and PM_10_ concentrations show a shift between peak hours during road transport rush hours (Table 9). In selected locations in Central Europe, shifts of ± 3 h have been reported in the morning and of up to + 5 h in the evening. In cities of South-Western Europe, this shift reached up to + 4 h in both the morning and in the evening. In the city of Thessaloniki (Greece) in South-Eastern Europe, there was a shift of ± 4 h in the morning and 0–3 h in the evening. Therefore, the “most and least favorable” ambient air-quality conditions were not strictly related/correlated to road traffic intensity in cities, towns, and villages. However, the “time shift” between emissions and increases/decreases in air pollutant concentrations could be the result of meteorological factors, which may (or may not) result in dispersal processes and coagulation, as well as in chemical and photochemical processes.

**Table 9:** Differences between the occurrence of maximum average hourly NO_2_ and PM_10_ concentrations and traffic rush hours (described as “traffic” stations)

Location, station type		Peak hour/difference (h)	Peak hour/difference (h)	Source	Description/period
Central Europe					
Hamburg	Traffic	08:00/-	17:00/-	Gaffron (2012)	Year
Warsaw, CB	NO_2_	–	22:00/+ 5	Menut et al. (2012)	Model, summer
	PM_10_	06:00/− 2	22:00/+ 5		
Poland, CB	NO_2_	09:00/+ 1	19:00/+ 2	Own analysis	Winter
	PM_10_	10:00/+ 2	21:00/+ 4		
	NO_2_	07:00/− 1	21:00/+ 4		Summer
	PM_10_	08:00/+ 0	22:00/+ 5		
Poland, TB	NO_2_	08:00/+ 0	19:00/+ 2		Winter
	PM_10_	09:00/+ 1	21:00/+ 4		
	NO_2_	05:00/− 3	21:00/+ 4		Summer
	PM_10_	06:00/− 2	21:00/+ 4		
Poland, RB	NO_2_	08:00/+ 2	19:00/+ 2		Winter
	PM_10_	09:00/+ 1	20:00/+ 3		
	NO_2_	–	22:00/+ 5		Summer
	PM_10_	06:00/− 2	19:00/+ 2		
South-West Europe					
Barcelona	Traffic	06:00/-	17:00/-	Pérez et al. (2010)	Summer, weekdays
Barcelona,CB	NO_2_	07/00/+ 1	21:00/+ 4		Summer, weekdays
	PM_1_	06:00/+ 0	21:00/+ 4		
	PM_1–2.5_	09:00/+ 3	–		
	PM_2.5–10_	10:00/+ 4	14:00/− 3		
Madrid, UT	NO_2_	08:00/+ 2	21:00/+ 4	Moreno et al. (2013)	Winter
	PM_10_	09:00/+ 3	20:00/+ 3		
South-East Europe					
Thessaloniki	Traffic	09:00/-	21:00/-	Vouitsis et al. (2015)	Winter
Thessaloniki, CB	PM_10_	13:00/+ 4	22:00/+ 1		Winter
	PM_10_	11:00/+ 2	21:00/+ 0		Summer
Thessaloniki, UT	NO_2_	13:00/+ 4	21:00/+ 0		Winter
	PM_10_	11:00/+ 2	24:00/+ 3		
	NO_2_	13:00/+ 4	21:00/+ 0		Summer
	PM_10_	07:00/− 2	21:00/+ 0		

Our study, based on Gaffron (2012), Menut et al. (2012), Pérez et al. (2010), Moreno et al. (2013), and Vouitsis et al. (2015)

*UT* air quality monitoring stations located near urban traffic

## Conclusions

The purpose of this investigation was to determine the “most” and ‘”least favorable” hours during the day in terms of ambient air quality for different types of settlement unit (cities, towns, and villages). The results could be used to help improve air quality management process in cities, towns, and villages. The concentrations of air pollutants were found to change dynamically during the day. Each air pollutant had a unusual, characteristic daily concentration profile, resembling a unimodal or bimodal distribution. The profiles changed significantly depending on the season. The concentrations of SO_2_, CO, PM_10_, PM_2.5_, and benzene were significantly higher in winter periods than during summer. The time between “peak hours” of air pollutant concentration during the day also was shorter in the winter.

The level of air pollutants was generally higher in the analyzed cities than in the towns and villages. Although the level of air pollution depended largely on the size of the settlement unit (city, town, or village), the daily profiles of the concentrations of air pollutants had relatively similar shapes. There were clear local maxima and minima during the day. The peak hours for air pollution usually did not coincide with the assumed traffic-intensity peaks (rush hours). This is in contrast to claims made in the literature that air pollution depends largely on road-traffic intensity (Agudelo-Castaneda and Teixeira 2014; Menut et al. 2012; Moreno et al. 2013, Pérez et al. 2010). Our analysis shows that there was a time shift of ± 5 h between the peak hours of air pollution and the transport rush hours. However, this time shift depended on the type of air pollutant, the season, and the size of the settlement unit.

Different approaches can be used to assess the daily changes in the concentration of pollution. It also is difficult to identify the most important air pollutants. The best and worst conditions in terms of ambient air quality should be determined individually for each country/region, depending on the pollutants, the climate, and the characteristics of the emission sources. Our analysis focused on high concentrations of NO_2_, CO, dust, and benzene, which were characterized by similar shapes of average hourly changes. Less attention was given to O_3_ and SO_2_. This was due to the fact that in the temperate climate of Central Europe, the ozone concentration in summer rarely exceeds permissible standards in cities and villages. Concentrations of sulphur dioxide in such areas are nowadays also very low. In Poland, this is a consequence of the modernization of the power generation sector (Cichowicz et al. 2017, 2018). However, permissible levels were exceeded in the cases of particulate matter (Wielgosiński et al. 2018; EEA 2016), benzene, and benzo(a)pyrene (EEA 2016) during winter periods. These air pollutants are related with the burning of fossil fuels for heating individual buildings.

Our analysis indicates that during winter the highest concentrations of pollutants occurred in cities, towns and villages in the mornings at 8:00–10:00 and evenings at 19:00–21:00 (local time). In summer periods, unfavorable conditions occurred in the mornings at 5:00–9:00 and in the evenings at 21:00–22:00 (local hour). The periods of worst air quality in the various settlement units differed by ± 2 h. The best ambient air-quality conditions in cities, towns, and villages, with the exception of ozone and taking into account mainly concentrations of NO_2_ and dust, occurred in the early mornings and in the afternoon. Therefore, the most favorable ambient air-quality conditions occurred between 3:00 and 5:00 and between 12:00 and 15:00 (local time). This information is particularly valuable for people to schedule outdoor activities, such as recreation or sport, as well as for structuring the working day (e.g., lunchtime, which usually takes place at 12:00–13:00) and time spent outside buildings. As a result, for example, children and the elderly may be recommended to engage in outdoor activities before 18:00 in urban areas during summer, because later in the day there is an increase in the concentrations of dust, NO_2_, and CO. However, there is a higher concentration of ozone at this time. Thus, air quality should be assessed on a case-by-case basis, for each area/region, taking into account both the type of settlement unit, its geographical location, the climate, and the types of emission sources.
